# Peptic Ulcer Disease Risk in Chronic Kidney Disease: Ten-Year Incidence, Ulcer Location, and Ulcerogenic Effect of Medications

**DOI:** 10.1371/journal.pone.0087952

**Published:** 2014-02-03

**Authors:** Chih-Chia Liang, Chih-Hsin Muo, I-Kuan Wang, Chiz-Tzung Chang, Che-Yi Chou, Jiung-Hsiun Liu, Tzung-Hai Yen, Chiu-Ching Huang, Chi-Jung Chung

**Affiliations:** 1 Division of Nephrology and Kidney Institute, China Medical University Hospital, Taichung, Taiwan; 2 Department of Medicine, College of Medicine, China Medical University, Taichung, Taiwan; 3 Department of Public Health, China Medical University, Taichung, Taiwan; 4 Management Office for Health Data, China Medical University Hospital, Taichung, Taiwan; 5 Department of Nephrology, Chang Gung Memorial Hospital, Taipei, Taiwan; 6 College of Medicine, Chang Gung University, Taoyuan, Taiwan; 7 Department of Medical Research, China Medical University Hospital, Taichung, Taiwan; 8 Department of Health Risk Management, College of Public Health, China Medical University, Taichung, Taiwan; Veterans Affairs Medical Center (111D), United States of America

## Abstract

**Objectives:**

We aimed at determining peptic ulcer disease (PUD) incidence among chronic kidney disease (CKD) patients during 1998–2008, compared to patients without CKD, and at examining associations between CKD and PUD.

**Methods:**

Data for 1998–2008 were extracted from the National Health Insurance Research Database in Taiwan. The annual PUD incidence (cases per thousand persons per year) was calculated separately for patients with and without CKD. Characteristics of patients with newly diagnosed PUD (n = 16322) were compared to those of a control group without PUD (n = 32644). The 2 groups were matched for age, sex, and index year. Odds ratios (OR) and 95% confidence intervals (CI) were estimated by logistic regression.

**Results:**

Over the 10-year period, the PUD incidence was ∼10–12 times higher in CKD patients than in those without CKD. Its incidence in elderly CKD patients increased rapidly over time. For CKD patients, most PUD events (>95%) were managed during hospitalization. Peptic ulcer risk, adjusted for all potential confounders, was much higher in CKD patients undergoing hemodialysis (adjusted OR, 9.74; 95% CI, 7.11–13.31). Maintenance hemodialysis patients were 2 times more likely to have gastric ulcers than duodenal ulcers, while CKD patients not on dialysis had similar risks for both. There were no significant interactions between medications and CKD status on the peptic ulcer risk. Unlike CKD patients on nonsteroidal anti-inflammatory drugs and clopidogrel, those on aspirin did not have a higher peptic ulcer risk (adjusted OR, 0.88; 95% CI, 0.44–1.77).

**Conclusions:**

CKD patients have a substantially increased PUD risk, and the majority of CKD patients with PUD require hospital management. Further, peptic ulcer risk is affected by hemodialysis therapy, patient status (inpatient vs. outpatient), and ulcerogenic medications.

## Introduction

Despite substantial advances in medicine, peptic ulcer disease (PUD) is still a common disease in elderly patients and patients with multiple comorbid conditions [1], [2]. Evidence suggests that *Helicobacter pylori* infection and use of non-steroidal anti-inflammatory drugs (NSAID) are the primary causes of PUD in the general population [1]. However, compared to the general population, patients with chronic kidney disease (CKD) have distinct causative factors and clinical outcomes of gastro-duodenal ulcers. Population-based studies have demonstrated that CKD patients have a higher risk of peptic ulcer bleeding and bleeding-related morbidity and mortality [3]–[5]. Tseng et al. reported a high recurrence rate of PUD among hemodialysis (HD) patients even after *H. pylori* eradication [6]. Another longitudinal study also reported that PUD occurred in a significant number of long-term HD patients despite a low prevalence of *H. pylori* infection [7].

Both PUD and CKD are leading public-health issues [8], [9], and many studies have described associations between them [2]–[7], [10], [11]. Despite this, limited information is available about temporal trends in PUD among CKD patients [5], [11]. Another limitation of existing evidence is the focus on hospitalized patients with peptic ulcer bleeding, preventing generalization to the entire CKD population [3], [4], [12]. It is also unclear if CKD patients differ from non-CKD peptic ulcer patients with respect to the ulcer location (gastric or duodenal mucosa) and patient status (inpatient or outpatient). Finally, it remains uncertain whether CKD patients taking ulcerogenic medications (e.g., NSAID or aspirin) are more likely to develop PUD.

With the aim of addressing these gaps in the literature, we conducted a population-based case-control study utilizing the Taiwan National Health Research Institute (NHRI) database to examine peptic ulcer risk among patients with CKD. In addition, we determined the incidence of PUD over a 10-year period, compared between CKD patients and patients without CKD. More specifically, the effects of gastroduodenal mucosa and ulcerogenic medications on CKD-related PUD were investigated.

## Methods

### Database

The Taiwan Bureau of National Health Insurance established the National Health Insurance Program in March 1995. It provides health care to more than 99% of the residents in Taiwan [13]. This population-based study utilized data from the National Health Insurance Research Database (NHIRD), which was established for research purposes. At the time of this study, it contained the claims data of 1 million randomly selected people from the total 23 million insured individuals registered from 1996 to 2008. There were no differences in age, sex, or medical costs between the database sample and the population insured under the National Health Insurance Program. The NHIRD data include sex, birthdate, dates of outpatient visits, dates of admission and discharge, surgical procedures, discharge diagnoses, and medication use. In this study, the International Classification of Diseases, Ninth Revision (ICD-9) codes were used to define diseases, medical procedures, and surgical procedures. Because the data were released for public access for research use, were anonymous, and were secondary, the study was exempt from full review by the Institutional Review Board.

### Study subjects

To determine the characteristics associated with PUD, we identified inpatient and ambulatory care patients with newly diagnosed peptic ulcers (ICD-9 codes 531, 532, and 533 for gastric ulcer [GU], duodenal ulcer [DU], and nonspecific peptic ulcer, respectively) between January 1, 1998, and December 31, 2008. An upper endoscopy (ICD-9 surgical codes: 41.1 and 45.1) was required to confirm the PUD diagnosis. To preclude non-specific kidney diseases from affecting the risk estimation, we defined CKD as a glomerular filtration rate <60 mL/min per 1.73 m^2^ for 3 months, i.e., chronic renal failure, which is compatible with the definition from the Kidney Disease Outcomes Quality Initiative (KDOQI) [14]. Therefore, CKD patients were chosen from the database using the ICD-9 diagnosis code 585 (chronic renal failure).

### Study design

The first part of the study included the entire insured population to compare the annual incidence of newly diagnosed peptic ulcers in CKD and non-CKD patients. The second part of this study was of a case-control design to examine associations between PUD and CKD. For each PUD case identified, 2 control patients without a history of PUD were randomly selected and matched for age (within 5 years), sex, and year of index date (date of the first PUD diagnosis). We excluded patients who were younger than 20 years of age, had a history of PUD, or underwent vagotomy or gastrectomy prior to January 1, 1998. This resulted in 16322 cases and 32644 controls.

### Data collection

The sociodemographic variables included sex, age, and residential area (urban/rural). The age of each patient was defined as the difference between the index date and the date of birth. In accordance with the National Statistics of Regional Standard Classification, all insured persons were grouped into 4 levels on the basis of population densities, with level 1 representing the highest urbanization and level 4 representing the lowest urbanization. Comorbidities were defined as diseases diagnosed on medical visits before the index date. All comorbid conditions were confirmed by at least 3 medical records. The comorbid conditions and corresponding ICD-9 codes identified for the full sample were as follows: hypertension (401.xx ∼ 405.xx, 642.xx), diabetes mellitus (250.xx), coronary artery disease (410.xx ∼ 414.xx), cerebrovascular disease (430.xx ∼ 437.xx), malignancy (140.xx ∼ 209.xx), liver cirrhosis (571.2x, 571.5x, 571.6x), chronic pulmonary disease (491.xx ∼ 505.xx), connective tissue disease (710.xx, 714.xx), and chronic kidney disease (585).

Dialysis treatment, including modality and length of treatment, was recorded. Because different dialysis modalities might influence ulcer risk, we excluded those who underwent peritoneal dialysis (39 patients) or renal transplantation (5 patients), resulting in 994 cases and 260 controls. The receipt of HD was identified by the ICD-9 treatment code 399.5 (hemodialysis). The CKD patients were specifically divided into the patients who had never received HD treatment (non-HD CKD) and the patients who had received HD treatment at any time, irrespective of the length of treatment (HD-CKD). The HD-CKD patients included patients who received HD for <3 months (temporary HD-CKD) and those who underwent HD for ≥3 months (maintenance HD-CKD) [15].

Depending on ulcer location and upper endoscopy timing (e.g., in outpatient department or during hospitalization), these groups were further stratified to observe the impact of CKD on the gastric and duodenal mucosa.

The *H. pylori*-associated peptic ulcer was identified by the receipt of *H. pylori* eradication therapy during or after the index date. *H. pylori* eradication therapy was defined as the administration of proton pump inhibitors or H2 receptor antagonists plus clarithromycin or metronidazole, plus amoxicillin or tetracycline, and with or without bismuth [16].

Medication use was documented for aspirin, NSAID (except aspirin), warfarin, clopidogrel, and cilostazol. NSAID consisted of cyclooxygenase-2-specific inhibitors (COXIBs) and traditional NSAID (except COXIBs). We identified medications using the National Drug Classification System and the Anatomic Therapeutic Chemical Code coordinated by the World Health Organization Collaborating Centre for Drug Statistics Methodology [17]. To investigate the effect of medication use on peptic ulcer risk, analysis was conducted with current users (use for more than three-quarters of the 90 days prior to the index date) and never users.

### Statistical analysis

The annual incidence of newly diagnosed peptic ulcers in CKD and non-CKD patients for the entire insured population was calculated and expressed as cases per thousand persons per year for the years 1998–2008. This was also implemented following stratification by CKD and sex and by CKD and age. Linear regression was conducted to determine the statistical significance.

Comparisons between the cases and controls for age, sex, urbanization, comorbidities, and medication use were conducted using logistic regression. The association between CKD and PUD was evaluated using unconditional logistic regression models to estimate the odds ratios (OR) and 95% confidence intervals (CIs) after adjustments for all potential confounders including age, sex, urbanization, comorbidities, CKD types (non-CKD vs. non-HD CKD vs. HD-CKD), patient status (inpatient vs. outpatient), and medication use. The significance of the trend and interaction for the different stratifications was tested using the same logistic regression model.

Analyses were performed using the SAS statistical software (version 9.1 for Windows; SAS Institute Inc., Cary, NC, USA). The results were considered statistically significant when the 2-tailed *p* values were less than 0.05.

## Results

### Temporal trends in the incidence of peptic ulcer disease

**Figure 1:**
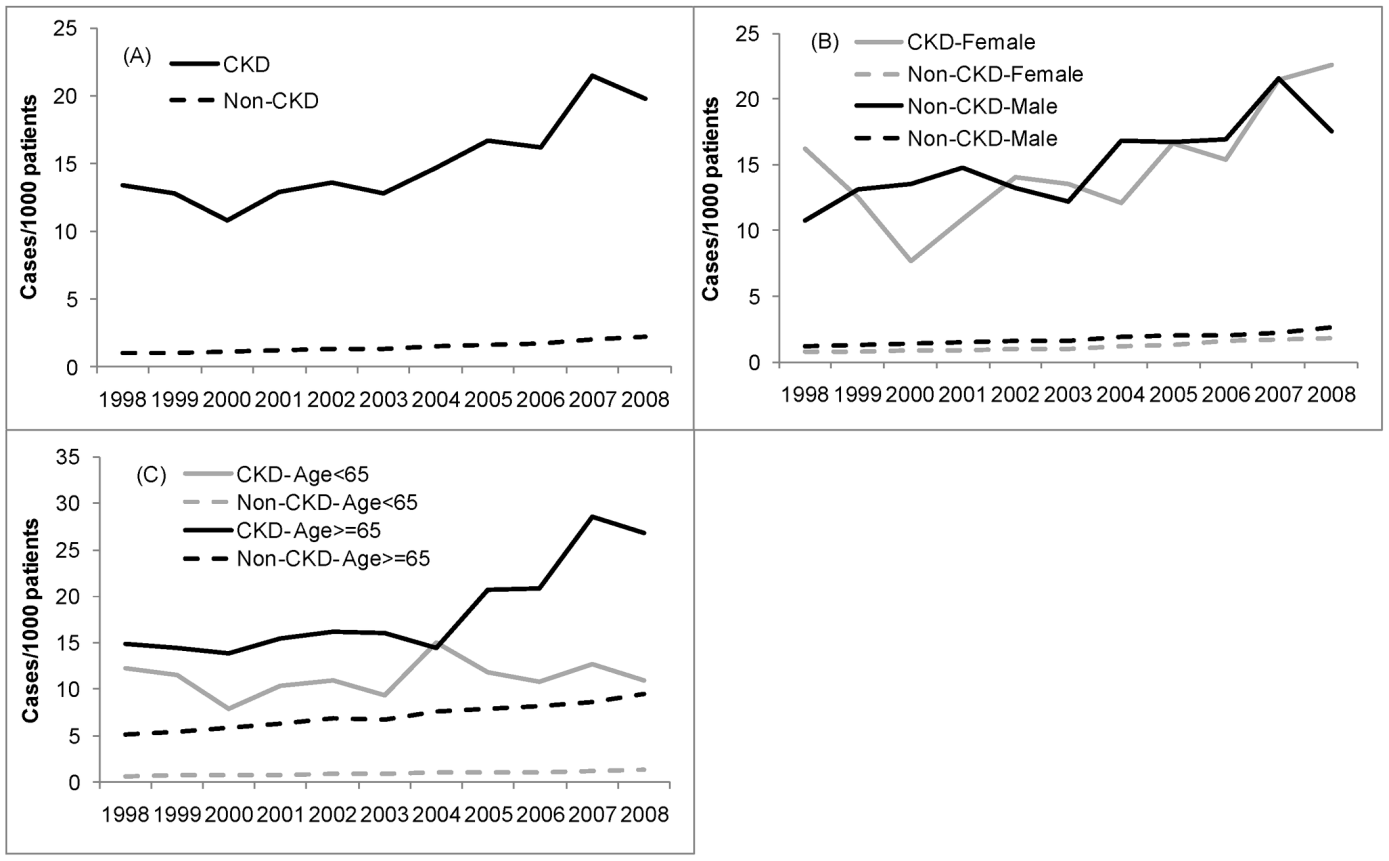
Incidence of peptic ulcer disease in patients registered in the National Health Insurance Research Database (NHIRD) of Taiwan for the years 1998–2008, stratified by (A) the presence of chronic kidney disease (CKD), (B) sex and presence of CKD, and (C) age and presence of CKD. The incidence is expressed as cases per 1000 persons per year.

The incidence of PUD was much higher in patients with CKD than in those without CKD (Figure 1A). In 1998, the incidence rates were 13.2/1000 persons in CKD and 1.1/1000 persons in non-CKD. This rate increased to 19.8/1000 persons in CKD patients in 2008 (*p* = 0.0009 for the trend), compared with 2.0/1000 persons in those without CKD (*p*<0.00001 for the trend). Throughout the study period, the incidence in CKD patients was approximately 10–12 times higher than in those without CKD (*p*<0.0001).

Figure 1B illustrates the incidence of PUD stratified by CKD and sex. There was no statistical difference in the incidence between genders (non-CKD *p* = 0.52; CKD *p* = 0.68). The results of the stratification for CKD and age are provided in Figure 1C. CKD patients aged ≥65 years had the highest incidence of PUD with a rapid increase following 2004. The incidence of PUD in non-CKD patients aged ≥65 years increased steadily between 1998 and 2008 (5.0/1000 persons and 9.1/1000 persons, respectively, *p* = 0.0002 for trend). The incidence of PUD in CKD patients aged <65 years slightly declined over time (12.5/1000 persons in 1998, 10.2/1000 persons in 2008, *p* = 0.52 for trend).

### Associations between peptic ulcer disease and clinical characteristics

Among the 16322 patients for whom the first episode of PUD occurred between 1998 and 2008, most were men (60.6%), and the mean age (SD) was 61.0 (17.1) years (Table 1). After adjustment for age and gender, the cases had significantly lower urbanization and more chronic diseases than controls. The highest peptic ulcer risk was noted in patients with liver cirrhosis (adjusted OR, 25.9; 95% CI, 21.8–30.8), followed by those with CKD (adjusted OR, 8.04; 95% CI, 7.00–9.23) (Table 1). The cases were more likely to be exposed to NSAID, warfarin, and clopidogrel than the controls, except for cilostazol. There were 648 patients receiving *H. pylori* eradication therapy who accounted for 3.97% of the cases.

**Table 1:** Sociodemographic and clinical characteristics of patients with peptic ulcer disease (cases) and patients without peptic ulcer disease (controls).

	N (%) of subjects		OR (95% CI)	
Characteristics	Controls (n = 32644)	Cases (n = 16322)	Crude	Adjusted†
**Age (years)**				
<40	4445 (13.6)	2223 (13.6)	1.00 (reference)	1.00 (reference)
40–49	4575 (14.0)	2287 (14.0)	1.00 (0.93–1.07)	1.00 (0.93–1.07)
50–59	5358 (16.4)	2679 (16.4)	1.00 (0.93–1.07)	1.00 (0.93–1.07)
60–69	6336 (19.4)	3168 (19.4)	1.00 (0.94–1.07)	1.00 (0.94–1.07)
70–79	7695 (23.6)	3848 (23.6)	1.00 (0.94–1.07)	1.00 (0.94–1.07)
≥80	4234 (13.0)	2117 (13.0)	1.00 (0.93–1.08)	1.00 (0.93–1.08)
Male	19792 (60.6)	9896 (60.6)	1.00 (0.96–1.04)	1.01 (0.97–1.05)
**Urbanization levels**				
1 (highest)	9853 (30.2)	3641 (22.3)	1.00 (reference)	1.00 (reference)
2	9022 (27.6)	4639 (28.4)	1.39 (1.32–1.47)	1.39 (1.32–1.47)
3	5702 (17.5)	2695 (16.5)	1.28 (1.21–1.36)	1.28 (1.21–1.36)
4 (lowest)	8065 (24.7)	5347 (32.8)	1.79 (1.70–1.89)	1.79 (1.78–1.88)
**Comorbidity**				
Hypertension	10826 (33.2)	8702 (53.3)	2.30 (2.21–2.39)	2.81 (2.69–2.94)
Diabetes mellitus	3713 (11.4)	3986 (24.4)	2.52 (2.40–2.65)	2.56 (2.43–2.69)
Coronary artery disease	4219 (12.9)	5205 (31.9)	3.15 (3.01–3.30)	3.53 (3.36–3.71)
Cerebrovascular disease	1416 (4.34)	2095 (12.8)	3.25 (3.03–3.48)	3.32 (3.09–3.57)
Malignancy	872 (2.67)	1131 (6.93)	2.71 (2.48–2.97)	2.69 (2.45–2.94)
Liver cirrhosis	139 (0.43)	1621 (9.93)	25.8 (21.7–30.7)	25.9 (21.8–30.8)
Chronic pulmonary disease	4322 (13.2)	5223 (32.0)	3.08 (2.95–3.23)	3.38 (3.22–3.54)
Connective tissue disease	1334 (4.09)	1859 (11.4)	3.02 (2.80–3.25)	3.02 (2.80–3.25)
Chronic kidney disease	260 (0.80)	994 (6.09)	8.07 (7.04–9.27)	8.04 (7.00–9.23)
*H. pylori*-associated peptic ulcer		648 (3.97)		
**Medication**				
NSAID	335 (1.03)	499 (3.06)	3.04 (2.64–3.49)	2.99 (2.60–3.45)
Aspirin	610 (1.87)	409 (2.51)	1.35 (1.19–1.53)	1.32 (1.16–1.50)
Warfarin	93 (0.28)	73 (0.45)	1.57 (1.16–2.14)	1.54 (1.13–2.10)
Clopidogrel	75 (0.23)	145 (0.89)	3.88 (2.94–5.14)	3.79 (2.86–5.01)
Cilostazol	24 (0.07)	20 (0.12)	1.67 (0.92–3.02)	—

† Adjusted model: adjusted for age and sex. Abbreviations: OR  = odds ratios; 95% CI  =  95% confidence interval; NSAID  =  nonsteroidal anti-inflammatory drug; NA  =  not applicable.

### Peptic ulcer risk estimation by H. pylori, CKD, and ulcer location

Table 2 provides the estimates of peptic ulcer risk between the different CKD types (non-CKD vs. non-HD CKD vs. HD-CKD) compared between different ulcer locations (GU vs. DU) and presence of *H. pylori* infection (yes vs. no). In general, CKD patients had an increased risk of PUD regardless of the receipt of HD or the presence of *H. pylori* infection, in comparison to those without CKD. Independent of *H. pylori* infection, HD-CKD patients had a higher risk for PUD than non-HD CKD patients. PUD was most likely to occur in HD-CKD patients on temporary HD (adjusted OR, 13.4; 95% CI, 5.09–35.0), followed by HD-CKD patients on maintenance HD (adjusted OR, 9.42; 95% CI, 6.76–13.1). Non-HD CKD patients had the lowest peptic ulcer risk (adjusted OR, 3.80; 95% CI, 3.19–4.52). The risk for GU was about 2-fold higher than that for DU for HD-CKD patients receiving maintenance HD (GU: adjusted OR, 11.6; 95% CI, 8.06–16.8; DU: adjusted OR, 5.44; 95% CI, 3.40–8.71), in contrast to similar risks for both ulcer locations in non-HD CKD patients (GU: adjusted OR, 4.23; 95% CI, 3.43–5.22; DU: adjusted OR, 4.07; 95% CI, 3.19–5.18).

**Table 2:** Adjusted odds ratios for the association between peptic ulcer disease and chronic kidney disease, stratified by Helicobacter pylori infection and ulcer location (duodenal ulcer and gastric ulcer).

	Peptic ulcer at any location									Specific ulcer location					
	*Total*			*H. pylori* (−)			*H. pylori* (+)			*Gastric ulcer*			*Duodenal ulcer*		
**CKD status**	N	Case	OR (95% CI)	N	Case	OR (95% CI)†	N	Case	OR (95% CI)†	N	Case	OR (95% CI)†	N	Case	OR (95% CI)†
Non-CKD^§^	47711	15328	1.00 (reference)	47086	14703	1.00 (reference)	33008	625	1.00 (reference)	37838	5455	1.00(reference)	35732	3349	1.00 (reference)
Non-HD CKD^#^	826	615	3.80 (3.19–4.52)	809	598	3.85 (3.23–4.58)	228	17	2.84 (1.70–4.73)	470	259	4.23 (3.43–5.22)	352	141	4.07 (3.19–5.18)
HD-CKD^&^	428	379	9.74 (7.11–13.3)	422	373	9.99 (7.29–13.7)	55	6	4.10 (1.74–9.70)	216	167	12.1 (8.52–17.1)	109	60	6.59 (4.31–10.1)
Temporary*	54	49	13.4 (5.09–35.0)	54	49	13.8 (5.24–36.2)	5	0	NA	24	19	15.9 (5.52–46.1)	20	15	16.3 (5.53–48.0)
Long-term^¶^	374	330	9.42 (6.76–13.1)	368	324	9.58 (6.87–13.4)	50	6	4.49 (1.89–10.7)	192	148	11.6 (8.06–16.8)	89	45	5.44 (3.40–8.71)

† Adjusted model: adjusted for age, sex, urbanization, comorbidity, medications, and patient status (inpatients vs. outpatients). ^§^Non- CKD: all patients without chronic kidney disease. ^#^Non-HD CKD: chronic kidney disease patients never undergoing any dialysis therapy. ^&^HD-CKD: chronic kidney disease patients ever receiving hemodialysis. *Temporary: chronic kidney disease patients receiving hemodialysis <3 months. ^¶^Long-term: chronic kidney disease patients undergoing hemodialysis ≥3 months. Abbreviations: OR  = odds ratios; 95% CI  =  95% confidence interval; CKD  =  chronic kidney disease; NA  =  not applicable.

### Peptic ulcer risk estimation by CKD, ulcer location, and patient status

Regarding patient status, most of the CKD patients were diagnosed with PUD during hospitalization, with 37 PUD diagnoses in CKD patients occurring in the ambulatory care department (Table 3). In the CKD patients, peptic ulcer risk was much higher among inpatients than among outpatients, irrespective of receiving HD. Inpatients on maintenance HD had a greater risk for GU than DU (GU, adjusted OR: 12.5; 95% CI, 8.61–18.0; DU, adjusted OR: 5.71; 95% CI, 3.54–9.20) compared to outpatients (GU, adjusted OR: 5.24; 95% CI, 1.75–15.7; DU, adjusted OR: 4.80; 95% CI, 1.10–20.8).

**Table 3:** Adjusted odds ratios for the association between peptic ulcer disease and chronic kidney disease, stratified by ulcer location (duodenal ulcer and gastric ulcer) and patient status (inpatient and outpatient)

		Peptic ulcer at any location			Gastric ulcer			Duodenal ulcer		
Patient status	CKD status	N	Case	OR (95% CI)†	N	Case	OR (95% CI)†	N	Case	OR (95% CI)†
Outpatient	Non-CKD^§^	34054	1671	1.00 (reference)	32855	472	1.00 (reference)	32662	279	1.00 (reference)
	Non-HD CKD^#^	232	21	1.78 (1.10–2.87)	220	9	2.63 (1.30–5.35)	216	5	2.66 (1.05–6.70)
	HD-CKD^&^	65	16	5.72 (3.08–10.6)	53	4	5.01 (1.68–14.9)	51	2	4.48 (1.04–19.3)
	Temporary*	5	0	NA	5	0	NA	5	0	NA
	Long-term^¶^	60	16	6.05 (3.23–11.3)	48	4	5.24 (1.75–15.7)	46	2	4.80 (1.10–20.8)
Inpatient	Non-CKD^§^	46040	13657	1.00 (reference)	37366	4983	1.00 (reference)	35453	3070	1.00 (reference)
	Non-HD CKD^#^	805	594	3.99 (3.35–4.76)	461	250	4.32 (3.49–5.34)	347	136	4.16 (3.25–5.31)
	HD-CKD^&^	412	363	10.3 (7354–14.2)	121	163	12.7 (8.96–18.0)	107	58	6.90 (4.49–10.6)
	Temporary*	54	49	14.6 (5.57–38.3)	24	19	17.3 (6.00–49.7)	20	15	18.1 (6.17–53.1)
	Long-term^¶^	358	314	9.97 (7.14–13.9)	188	144	12.5 (8.61–18.0)	87	43	5.71 (3.54–9.20)

† Adjusted model: adjusted for age, sex, urbanization, comorbidity, and medications. ^§^Non- CKD: all patients without chronic kidney disease. ^#^Non-HD CKD: chronic kidney disease patients never undergoing any dialysis therapy. ^&^HD-CKD: chronic kidney disease patients ever receiving hemodialysis. *Temporary: chronic kidney disease patients receiving hemodialysis <3 months. ^¶^Long-term: chronic kidney disease patients undergoing hemodialysis ≥3 months. Abbreviations: OR  = odds ratios; 95% CI  =  95% confidence interval; CKD  =  chronic kidney disease; NA  =  not applicable.

### Peptic ulcer risk estimation in CKD patients receiving ulcerogenic medications

In the patients not receiving medications, the peptic ulcer risks in the non-HD CKD patients and HD-CKD patients were approximately 4.0 times and 9.4 times higher than that in the non-CKD patients, respectively. In the patients receiving NSAID, the peptic ulcer risk was 4.6 times higher in the non-HD CKD patients than in the non-CKD patients. Conversely, the CKD patients receiving aspirin, warfarin, clopidogrel, or cilostazol did not have a higher risk for PUD, in comparison to the non-CKD patients receiving aspirin (Table 4). The results of the analysis for the trends indicated the combined factors of CKD and NSAID or CKD and clopidogrel obviously increased the peptic ulcer risk. In contrast to the effect of NSAID or clopidogrel, CKD patients receiving aspirin did not have a higher peptic ulcer risk (adjusted OR: 0.88, 95% CI, 0.44–1.77) (Table 5). No significant interactions were found between each of the medications and CKD status on the peptic ulcer risk.

**Table 4:** The association between peptic ulcer disease and chronic kidney disease, stratified by medication use.

		N (%) of subjects		OR (95% CI)	
Medication use	CKD status	Controls (n = 32644)	Cases (n = 16322)	Model 1	Model 2
No use of any drugs	Non-CKD^§^	31323 (99.3)	14338 (94.1)	1.00 (reference)	—
	Non-HD CKD^#^	185 (0.59)	557 (3.65)	4.03 (3.35–4.85)	—
	HD-CKD^&^	48 (0.15)	349 (2.29)	9.44 (6.86–12.99)	—
NSAID	Non-CKD^§^	331 (98.8)	461 (92.4)	1.00 (reference)	1.00 (reference)
	Non-HD CKD^#^	4 (1.19)	24 (4.81)	4.39 (1.37–14.03)	4.62 (1.40–15.26)
	HD-CKD^&^	0 (0.00)	14 (2.81)	NA	NA
Aspirin	Non-CKD^§^	593 (97.2)	376 (91.9)	1.00 (reference)	1.00 (reference)
	Non-HD CKD^#^	17 (2.79)	23 (5.62)	1.80 (0.88–3.68)	1.77 (0.86–3.63)
	HD-CKD^&^	0 (0.00)	10 (2.44)	NA	NA
Warfarin	Non-CKD^§^	90 (96.8)	65 (89.0)	1.00 (reference)	1.00 (reference)
	Non-HD CKD^#^	2 (2.15)	5 (6.85)	4.71 (0.73–30.24)	4.33 (0.63–29.72)
	HD-CKD^&^	1 (1.08)	3 (4.11)	2.65 (0.22–32.51)	2.18 (0.16–29.72)
Clopidogrel	Non-CKD^§^	73 (97.3)	131 (90.3)	1.00 (reference)	1.00 (reference)
	Non-HD CKD^#^	2 (2.67)	10 (6.90)	3.27 (0.62–17.35)	3.15 (0.59–16.79)
	HD-CKD^&^	0 (0.00)	4 (2.76)	NA	NA
Cilostazol	Non-CKD^§^	22 (91.7)	18 (90.0)	1.00 (reference)	1.00 (reference)
	Non-HD CKD^#^	2 (8.33)	1 (5.00)	0.24 (0.01–8.37)	0.24 (0.01–11.34)
	HD-CKD^&^	0 (0.00)	1 (5.00)	NA	NA

Model 1: adjusted for age, sex, urbanization, and comorbidities. Model 2: adjusted for age, sex, urbanization, comorbidities, patient status (inpatient vs. outpatient), and other medications listed. ^§^Non- CKD: all patients without chronic kidney disease. ^#^Non-HD CKD: chronic kidney disease patients never undergoing any dialysis therapy. ^&^HD CKD: chronic kidney disease patients ever receiving hemodialysis. Abbreviations: NA  =  not applicable; NSAID  =  non-steroidal anti-inflammatory drug; CKD  =  chronic kidney disease; OR  = odds ratios; 95% CI  =  95% confidence interval.

**Table 5:** The effect of interaction between chronic kidney disease status and medication use on peptic ulcer risk.

CKD Status	Medication Use	Number of cases/Population	Adjusted OR (95% CI)†	p value for trend†	p value for interaction†
CKD	Aspirin			<0.0001	0.07
*No*	*No*	14952/46742	1.00 (reference)		
	*Yes*	376/969	0.67 (0.57–0.77)		
*Yes*	*No*	961/1204	2.52 (1.84–3.45)		
	*Yes*	33/50	0.88 (0.44–1.77)		
CKD	NSAID			<0.0001	0.67
*No*	*No*	14867/46919	1.00 (reference)		
	*Yes*	461/792	1.92 (1.64–2.25)		
*Yes*	*No*	956/1212	9.35 (6.71–13.0)		
	*Yes*	38/42	19.6 (6.24–61.6)		
CKD	Warfarin			<0.0001	0.70
*No*	*No*	15263/47556	1.00 (reference)		
	*Yes*	986/1243	0.86 (0.61–1.23)		
*Yes*	*No*	65/155	2.41 (1.77–3.28)		
	*Yes*	8/11	1.53 (0.34–6.93)		
CKD	Clopidogrel			<0.0001	0.88
*No*	*No*	15197/47507	1.00 (reference)		
	*Yes*	980/1238	1.91 (1.40–2.61)		
*Yes*	*No*	131/204	2.39 (1.75–3.27)		
	*Yes*	14/16	5.19 (1.08–25.0)		
CKD	Cilostazol			<0.0001	0.12
*No*	*No*	15310/47671	1.00 (reference)		
	*Yes*	992/1250	0.52 (0.26–1.04)		
*Yes*	*No*	18/40	2.42 (1.78–3.30)		
	*Yes*	2/4	0.41 (0.05–3.77)		

† Adjusted model: adjusted for age, sex, urbanization, comorbidities, CKD types (non-CKD vs. non-HD CKD vs. HD-CKD), patient status (inpatient vs. outpatient), and other medications listed. Abbreviations: NSAID  =  non-steroidal anti-inflammatory drug; CKD  =  chronic kidney disease.

## Discussion

In this nationwide population-based study, we investigated the incidence of PUD in the general population and in patients with CKD over a 10-year period. Our results indicated an incidence of PUD in the general population between 1.1 and 2.0 per 1000 persons per year, which reflects the global incidence [18]. The incidence in patients with CKD increased from 13.2 to 19.8 per 1000 persons/year over that time, and the incidence was 10–12 times higher than in patients without CKD. More importantly, there was a rapid increase in the incidence of PUD in elderly patients with CKD, compared to a decrease in younger CKD patients. Several factors, including HD therapy, patient access (inpatient vs. outpatient), and the use of NSAID and clopidogrel, further affected peptic ulcer risk in CKD patients. In addition, CKD patients undergoing maintenance HD were likely to develop GU following long-term HD therapy. Overall, we suggest that CKD itself is a strong independent risk factor for PUD, and the incidence of PUD among elderly CKD patients is substantially increasing.

Well-developed studies have demonstrated that CKD patients with peptic ulcer bleeding were more likely to have adverse outcomes such as prolonged hospital days and increased mortality rates [4], [12]. Since CKD is an important public issue not only in Taiwan but also worldwide [8], understanding the associated burden and actively promoting prevention become more urgent. Two large-scale epidemiological studies utilizing the US Renal Data System (USRDS) database documented an incidence of PUD-related bleeding among ESRD patients between 12.3 and 22.1 per 1000 persons per year [5], [11]. Similarly, we reported an incidence of PUD among CKD patients between 13.2 and 19.8 per 1000 persons per year. The incidence of PUD in the general population is reported to be decreasing [18], [19], while our results indicate that the incidence is increasing in patients with CKD. The decrease in PUDs in the general population may be the result of a decrease in *H. pylori* infections [18]–[20]. Given that the prevalence of *H. pylori* infection in CKD patients is lower than in those with normal renal function [7], the increase in PUD in this population is likely due to other causes. In recent decades, CKD has been associated with increasing age, the presence of a greater number of comorbidities, and an increased use of NSAID [21], [22]. Therefore, we thought that these factors are probable explanations for this increasing incidence of PUD in CKD, particularly as they are also well-known risk factors for PUD [1].

Our results indicate a deleterious influence of HD therapy on peptic ulcer risk, regardless of dialysis duration. Anticoagulant use during HD may contribute to this risk [3]–[6], [10]–[12]. In addition, intradialytic hypotension and hemodynamic changes might play a role in the occurrence of PUD. Intradialytic hypotension remains one of the most common HD problems and occurs in approximately 20–30% of HD sessions [23]. Because hypotension induces splanchnic hypoperfusion and subsequent gastrointestinal mucosal ischemia, stress ulcer-like mucosal lesions are very likely to occur [24]. Moreover, acute illness, such as sepsis or acute myocardial infarction, leads to more frequent hypotension during HD [23]. It may be speculated that acute illness, and therefore intradialytic hypotension, is more likely to occur in CKD patients receiving temporary HD. This might explain the increased peptic ulcer risk in CKD patients receiving temporary HD compared to those on maintenance HD.

It has been reported that the incidence of DU is higher than that of GU in the general population [20], [25]. In the current study, the occurrence of GU and DU was similar in the non-HD CKD patients, while GU occurred twice as often as DU among those undergoing maintenance HD. These results support several previous studies that have mentioned a higher rate of GU than DU among dialysis patients [6], [7], [11]. We suggest that impaired gastric emptying related to declining renal function may explain this phenomenon [26]. Accelerated gastric emptying contributes to the risk of DU [27]; therefore, impaired gastric emptying related to uremia may damage gastric mucosa and increase the risk of GU [26], [27].

The majority of CKD cases (96.3%) in this study received endoscopic diagnoses and treatments in the inpatient setting instead of the outpatient department. Yang et al indicated that approximately 10% of upper gastrointestinal bleeding episodes in the dialysis population were managed in the ambulatory care department [5]. It should be noted that PUD may be the primary cause of admission or may be a complication in those already hospitalized for another condition [5], [28]. Even though CKD patients are considered to have a high risk for ulcer rebleeding and require more surveillance in hospitals [4], [12], this heightened awareness does not fully explain this result. We propose that universal health insurance in Taiwan may explain the greater number of hospitalizations, due to the accessibility and affordability of care [29].

Similar to previous findings, warfarin was not associated with an increased risk for PUD in the current study [3], [11], [30]–[32]. However, clopidogrel increased the peptic ulcer risk in the patients, regardless of CKD status. A growing body of evidence indicates that clopidogrel alone is related to a higher rate of recurrent ulcer bleeding in high-risk patients, compared to the combination of aspirin and esomeprazole or clopidogrel and esomeprazole [33], [34]. Although the independent effect of clopidogrel on the risk for PUD remains uncertain, clopidogrel may impair ulcer healing by blocking the adenosine diphosphate receptor and subsequently contributing to the progression of underlying erosions to symptomatic ulcers [35].

Unexpectedly, the peptic ulcer risk in CKD patients on aspirin was quite distinct from that in CKD patients on NSAID. The use of aspirin did not increase the risk for PUD, which seems contradictory to the well-documented increase in peptic ulcer risk. However, this corresponding result had been also described in patients with diabetes [36], chronic obstructive pulmonary disease [30], and osteoporosis [38] in addition to those receiving peritoneal dialysis [10] and HD [3], [37]. There are several potential explanations. First, the use of aspirin usually leads to more limited gastroduodenal injuries (e.g., erosions) than conventional NSAID [32]. Because our study confirmed PUD using both clinical and endoscopic diagnoses, subclinical aspirin-associated erosion may have been missed. Second, physicians may only prescribe aspirin for patients with a lower risk for PUD, such as younger patients or those with few comorbid conditions, potentially resulting in bias. Last, despite adjusting for potential confounders, bias may be inevitable in observational studies owing to underreporting aspirin use related to its availability without a prescription [39].

Our study has several strengths. We utilized a national population database, minimizing selection bias and providing sufficient statistical power. The inclusion of inpatients and outpatient as well as PUD with and without bleeding allows for generalization to clinical practice for general CKD patients. Most importantly, the present study examined the risk for PUD and not upper gastrointestinal bleeding (UGIB). UGIB consists of many diseases, which presents challenges in conducting appropriate and meaningful analyses.

In spite of the listed strengths, certain limitations exist in this study. First, the NHIR Database does not provide relevant laboratory values and data on lifestyle risk factors such as smoking and alcohol consumption. Second, we did not have the information about the location of the gastric ulcer (e.g., cardia or pylorus). However, we consider that this limitation would not affect the overall risk estimation of PUD. Third, the data regarding *H. pylori* infection in this study were obtained indirectly by noting the receipt of *H. pylori* eradication therapy. Therefore, these results should be interpreted cautiously. Last, we did not collect data on proton pump inhibitors (PPI) or histamine 2 receptor antagonists (H2RA) to assess their effects on peptic ulcer risk. This information was not provided in the database because they are not prescribed for prophylactic purpose by decree of the National Health Insurance [3]. Further studies might be warranted to investigate the effects of the above factors on the association between CKD and PUD.

## Conclusions

In conclusion, we found that the incidence of PUD was more than 10 times higher in CKD patients than in those without CKD over a 10-year period between 1998 and 2008. CKD patients receiving HD, NSAID, or clopidogrel had an increased risk of PUD, compared to CKD patients not receiving these treatments. Peptic ulcer risk might be influenced by ulcer location, HD therapy, inpatient status, and ulcerogenic medications. Given the public health impact of both conditions, more research relating to peptic ulcer risk in the CKD population is warranted.
